# Use of intra-osseous access in adults: a systematic review

**DOI:** 10.1186/s13054-016-1277-6

**Published:** 2016-04-14

**Authors:** F. Petitpas, J. Guenezan, T. Vendeuvre, M. Scepi, D. Oriot, O. Mimoz

**Affiliations:** Department of Anesthesiology and Intensive Care, University Hospital of Poitiers, 86021 Poitiers, France; Laboratory of Anatomy, Biomechanics and Simulation, University Hospital of Poitiers, 86021 Poitiers, France; Emergency Department, University Hospital of Poitiers, 86021 Poitiers, France; Orthopedic Surgical Department, University Hospital of Poitiers, 86021 Poitiers, France; Pediatric Emergency Department, University Hospital of Poitiers, 86021 Poitiers, France

**Keywords:** Emergency, Intensive care unit, Safety, Skill acquisition

## Abstract

**Background:**

Indications for intra-osseous (IO) infusion are increasing in adults requiring administration of fluids and medications during initial resuscitation. However, this route is rarely used nowadays due to a lack of knowlegde and training. We reviewed the current evidence for its use in adults requiring resuscitative procedures, the contraindications of the technique, and modalities for catheter implementation and skill acquisition.

**Methods:**

A PubMed search for all articles published up to December 2015 was performed by using the terms “Intra-osseous” AND “Adult”. Additional articles were included by using the “related citations” feature of PubMed or checking references of selected articles. Editorials, comments and case reports were excluded. Abstracts of all the articles that the search yielded were independently screened for eligibility by two authors and included in the analysis after mutual consensus. In total, 84 full-text articles were reviewed and 49 of these were useful for answering the following question “when, how, and for which population should an IO infusion be used in adults” were selected to prepare independent drafts. Once this step had been completed, all authors met, reviewed the drafts together, resolved disagreements by consensus with all the authors, and decided on the final version.

**Results:**

IO infusion should be implemented in all critical situations when peripheral venous access is not easily obtainable. Contraindications are few and complications are uncommon, most of the time bound to prolonged use. The IO infusion allows for blood sampling and administration of virtually all types of fluids and medications including vasopressors, with a bioavailability close to the intravenous route. Unfortunately, IO infusion remains underused in adults even though learning the technique is rapid and easy.

**Conclusions:**

Indications for IO infusion use in adults requiring urgent parenteral access and having difficult intravenous access are increasing. Physicians working in emergency departments or intensive care units should learn the procedures for catheter insertion and maintenance, the contraindications of the technique, and the possibilities this access offers.

**Electronic supplementary material:**

The online version of this article (doi:10.1186/s13054-016-1277-6) contains supplementary material, which is available to authorized users.

## Background

In patients experiencing shock, severe dehydration, cardiac arrest, major trauma or airway compromise, and having difficult peripheral intravenous (IV) access because of edema, obesity, burns, medical history of IV drug abuse or others, physicians have three choices to administer fluids and medication during initial resuscitation: insertion of a central venous catheter, insertion of an ultrasound-guided peripheral venous catheter [1], or placement of an intra-osseous (IO) device. In this setting, procedure for IO infusion is shorter and has a higher success rate on first attempt than other routes [2, 3]. Moreover, in patients experiencing cardiac arrest, the procedure does not require stopping cardiopulmonary resuscitation and therefore may improve patient survival.

IO infusion is a rapid and safe method for obtaining parenteral access in patients with difficult venous access. Pioneered in 1922 by Drinker and colleagues [4], this access was not used to benefit peripheral IV until the 1980s. Pediatricians have used this route for three decades in frequent emergency situations like hypovolemic shock in dehydrated infants. In adults, IO access is less frequently used. However, it has been recommended for about 10 years in the case of failure of peripheral IV line placement during resuscitation [5]. Unfortunately, physicians do not frequently perform this procedure despite the availability of new mechanical devices with high success rate after brief training [6]. Yet, IO access is the quickest way to establish access for rapid infusion of fluids, drugs, and blood products in emergency situations as well as for cardiac resuscitation [7].

The aim of this systematic review is to summarize the most relevant information on IO infusion in adults to promote its use by physicians working in intensive care units and emergency departments.

## Methods

**Fig. 1:**
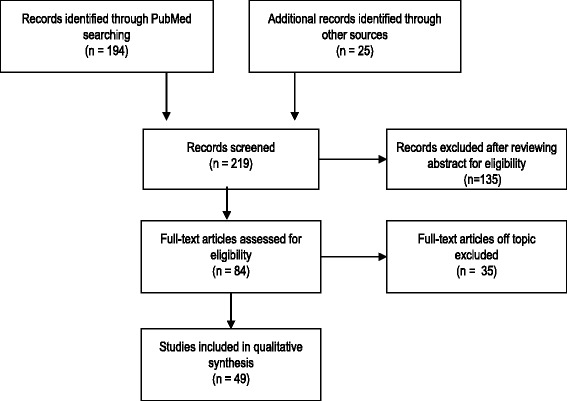
Flow diagram of studies selection

A PubMed search for all articles published up to December 2015 was performed by using the terms “Intra-osseous” AND “Adult” (Title/Abstract), which returned 194 records (Fig. 1). Retrieval was not limited in time, but only manuscripts in English or French were selected. Editorials, comments, and case reports were excluded. Additional articles were included by using the “related citations” feature of PubMed. The references of the included articles were reviewed to ensure no relevant articles had been missed. Finally, the most recent reviews or guidelines on IO or peripheral IV infusion were searched. In total, 25 more records were added.

Abstracts of the 219 articles that the search yielded were reviewed independently by two authors (FP and JG) and 135 of them out of scope of this review or concerning only children were excluded. The same two authors independently reviewed the full text of the remaining 84 records to select those useful for answering the following question: when, how, and for which population should an IO infusion be used in adults. Finally, a total of 49 records were retained by mutual consensus, and pertinent data were extracted to prepare independent drafts. Once this step had been completed, all authors met, reviewed the drafts together, resolved disagreements by consensus with all the authors, and decided on the final version.

## Results and Discussion

### Historical background and recommendations for IO access use

In 1922, Drinker described the marrow of a mammalian bone as a “non-collapsible vein” [4]. In 1934, Josefson used for the first time an IO access in humans via sternal location to administer liver concentrate in patients with pernicious anemia [8]. Then, in 1940, Tocantins and O’Neill described successful administration of blood, saline solutions and glucose using an IO access in pediatric patients [9]. IO access was then forgotten by physicians and reappeared in the 1980s for children only. It took another two decades before recommendations for IO access were included in the 2005 American Heart Association guidelines for cardiopulmonary resuscitation and emergency cardiovascular care for adults [10]. Their last guidelines published in 2015 highlighted the place of IO access when IV access is not readily available in adults [5]. Moreover, it is pointed out that IO or IV drug administration should be preferred to endotracheal administration. Indeed, IO access allows for fluid resuscitation as well as high-volume drugs with efficacy similar to an IV access. By contrast, endotracheal administration allows only specific drugs that have low toxicity to lung tissue, and their bioavailability is more variable [5]. In trauma patients, resuscitation often needs the administration of large volume of fluids. A large-caliber peripheral IV catheter may be preferred, although its insertion may be more difficult. Similarly, the tactical combat casualty care guidelines recommend the use of IO access when resuscitation is required and IV access is not easily obtainable [11]. In extension, there is evidence that IO access should be used in all adult trauma patients when an IV catheter is not readily available, despite lack of randomized studies.

Despite these guidelines, IO access remains largely underused. In the UK, only 7 % of 157 physicians working in accident and emergency departments and seeing more than 30,000 new patients per year used IO access in 2000, while 74 % were aware that it could be used in adult patients [12]. More recently, in Denmark, 23.5 % of 759 physicians responding to a questionnaire were aware that they could use IO access in their practice, but none of them did because of the lack of training and equipment [13]. In 2009, in the United States, 72 % of national emergency medicine programs strongly recommended the use of IO access in adult patients [14]. Despite this recommendation, only 73 IO accesses were placed in 3847 unstable patients with unobtainable peripheral IV access in the year prior to the survey. Sixty-two percent of physicians who had failed in peripheral vascular access chose central line access for the second attempt. If a third attempt was required, central venous access remained the predominant choice. IO access became the technique of choice only if a fourth attempt was required.

### Anatomy

When direct vascular access is difficult, the anatomical characteristics of bone make IO access the most interesting way for vascular access to start treatment of shock. Indeed, the highly vascularized bone marrow is connected to the central vascular system via the medullary venous channel to nutrient and emissary veins. The hardness of compact bone and the presence of bone spicules where the marrow is contained make this cavity a non-collapsible system even in the presence of shock or profound hypovolemia [1]. Despite the fact the cavity is empty after saline flush, bone spicules increase flow resistance between bone cavity and vascular system. Therefore, use of pressure bags is absolutely required to enhance flow rates [15] and enable medications to reach the vascular system. When this recommendation is implemented, the IO access allows rapid delivery of large amounts of fluids and medications in emergency situations.

### Indications

In adults, IO access is required in emergency situations as soon as peripheral access is not easily obtainable. It can be used for drug administration, fluid perfusion infusion, and to draw blood samples. The most frequent clinical situations needing IO access in adult patients remain cardiopulmonary resuscitation for epinephrine administration [5], and trauma for the easiness of access [16].

### Contraindications

The main contraindications for IO access are summarized in Table 1. As regards all vascular accesses, infection at insertion site should lead to the choice of an alternative site to avoid spreading sepsis or osteitis. A fractured bone leads directly to extravasation of fluids and infused medications and thus to complete inefficacy of IO access. In a general manner, IO access should not be used in severe genetic or acquired bone diseases, imperfect osteogenesis, osteoporosis and osteomyelitis [17].

**Table 1:** Main contraindications of intra-osseous access

Skin infection at insertion site
Fractured bone
Severe bone diseases
Imperfect osteogenesis
Osteoporosis
Osteomyelitis
Compartment syndrome in target extremity
Prior surgery
Burns
Localized cellulitis at device insertion site
Recent failed intra-osseous attempt in same bone

### IO infusion devices

**Fig. 2:**
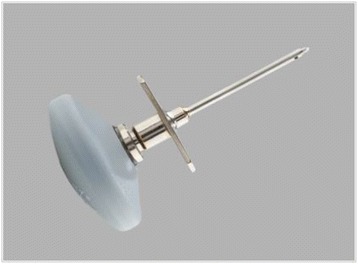
Dieckmann modified needle

**Fig. 3:**
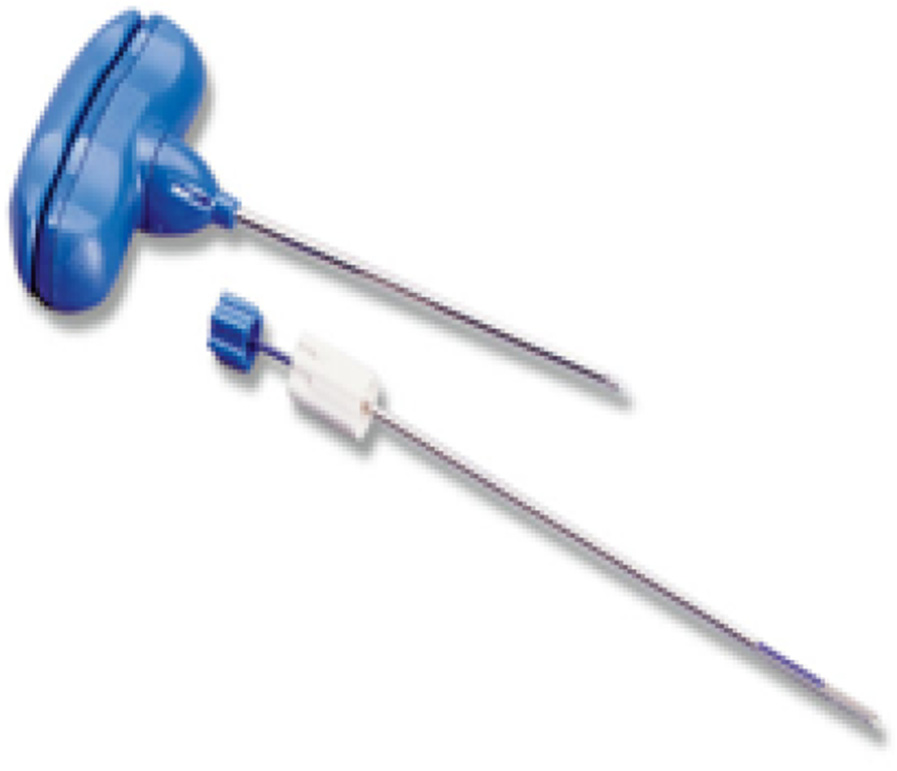
Jamshidi needle

Manual and semi-automatic devices are available for IO access. Manual devices require a specific needle with a central removal stylet. The most commonly used devices are the Dieckmann modified needle (Fig. 2) with two opposing side ports at the tip to promote unobstructed flow (Cook Medical Incorporation, Bloomington, IN, USA) and the Jamshidi needle (Fig. 3) for bone marrow biopsy (Cardinal Health, Dublin, OH, USA). These devices are easier to use in young children than in adults.

**Fig. 4:**
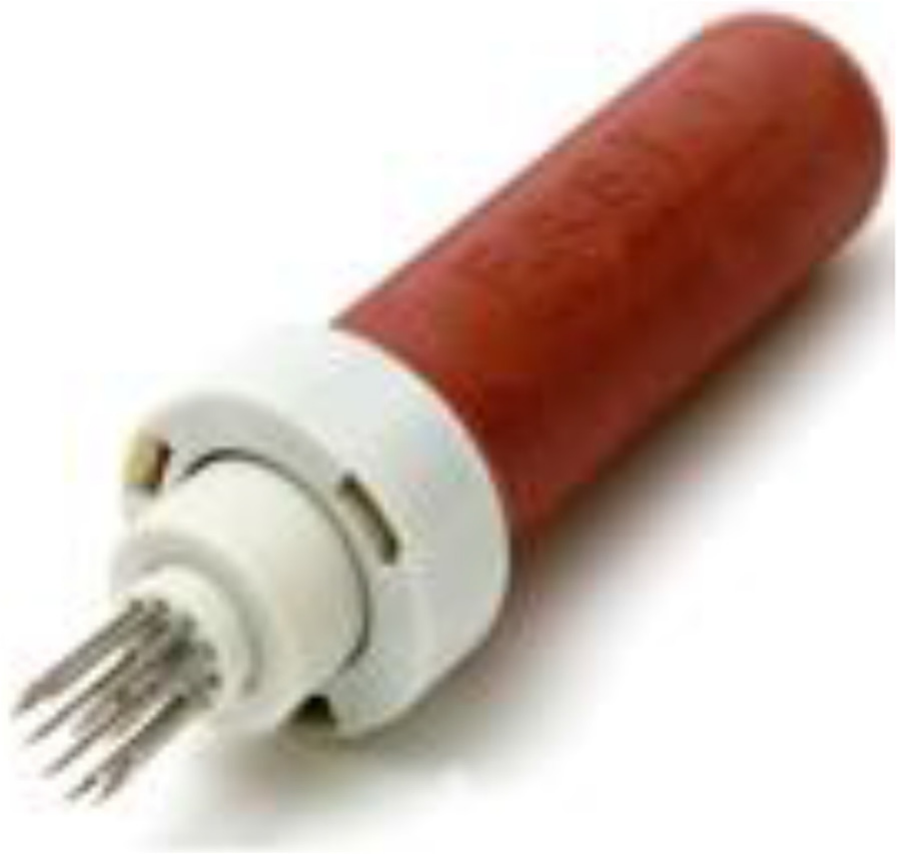
FAST1® device

**Fig. 5:**
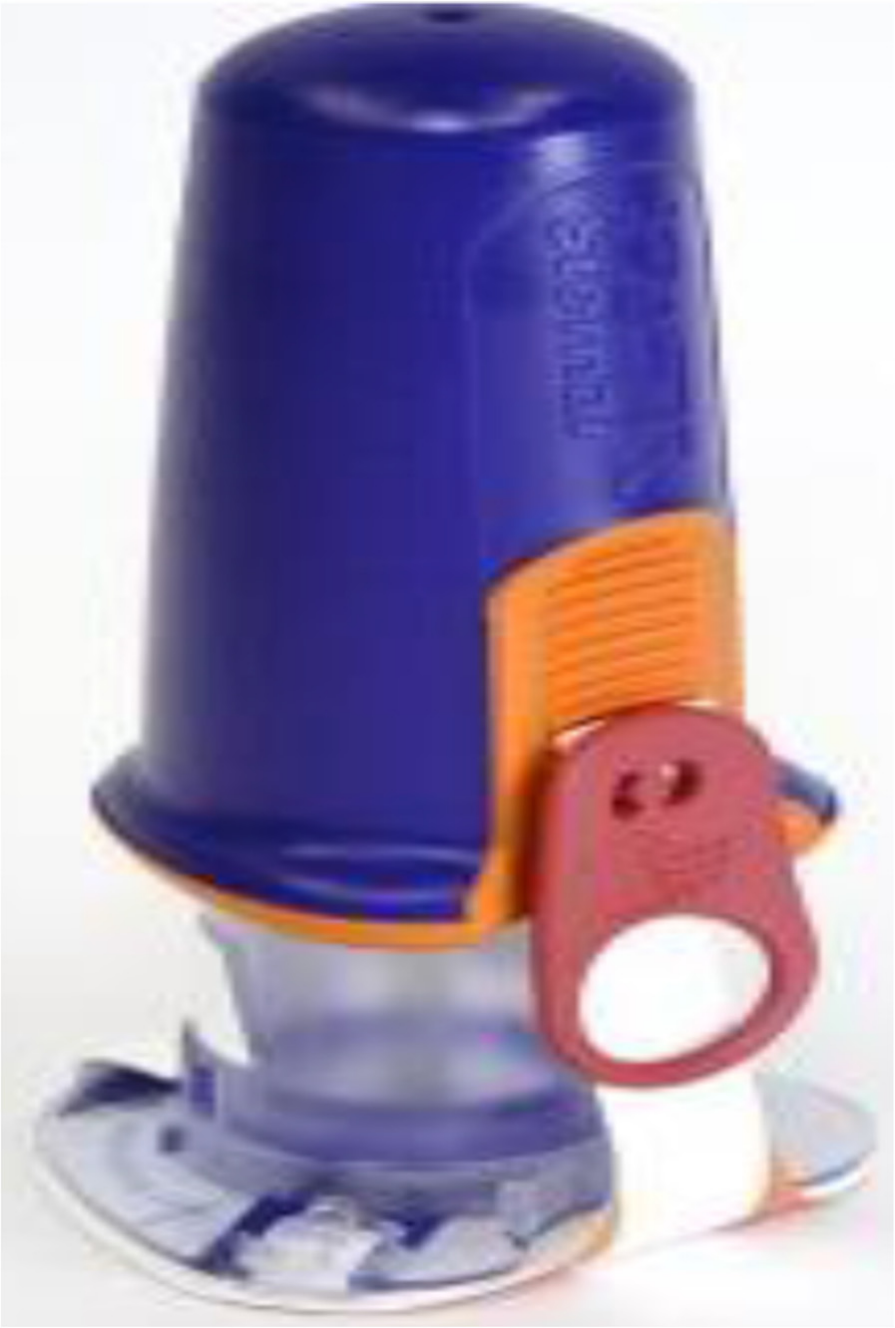
FASTx® device

**Fig. 6:**
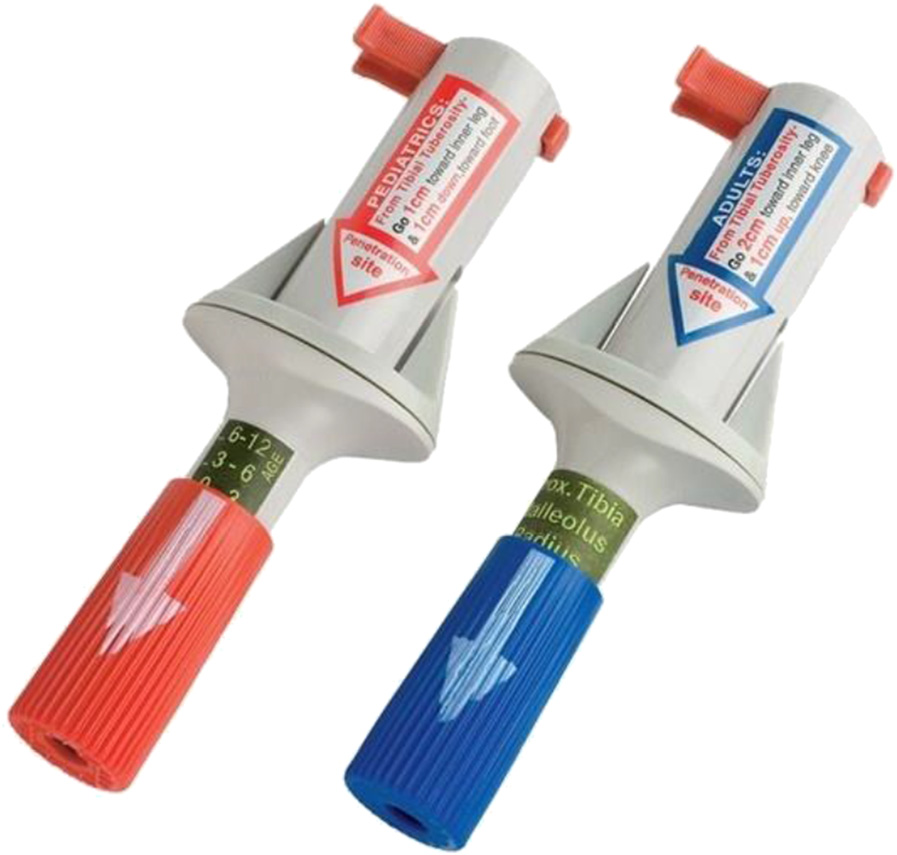
BIG® device

**Fig. 7:**
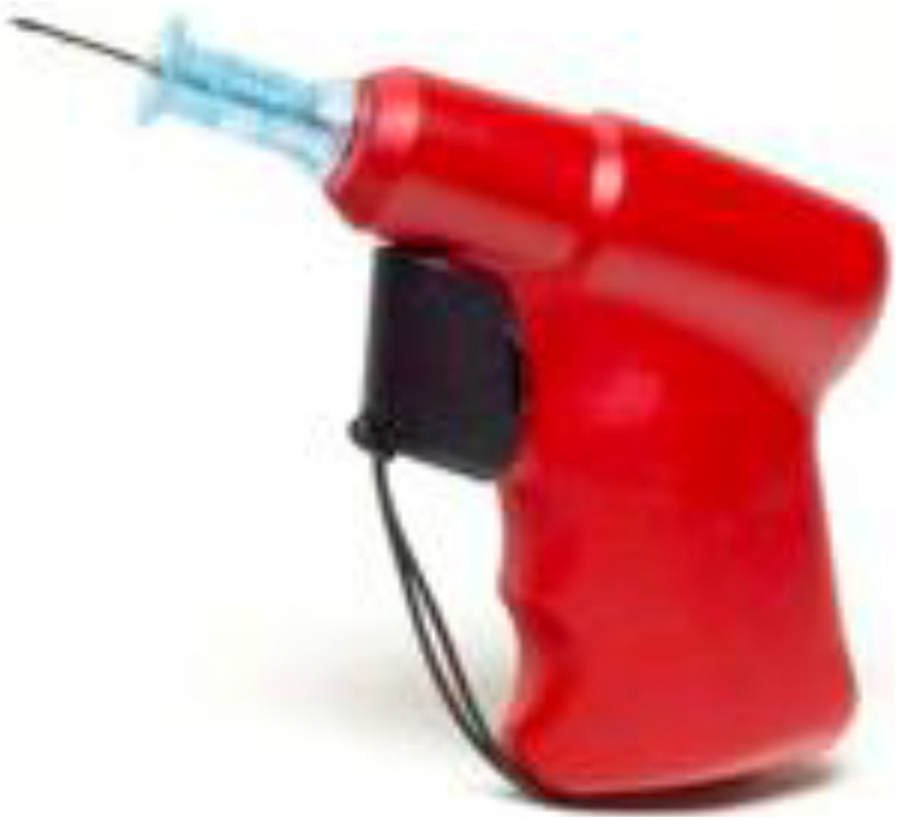
EZ-IO® device

Semi-automatic devices are preferred in adults despite the lack of data proving their superiority. Three types of devices are available. Two are disposable – the FAST1® (Fig. 4) and since 2010 the FASTx® (Fig. 5) (Pyng Medical Corporation, Vancouver, BC, Canada) for sternal insertion only, and the Bone Injection Gun (BIG®) (Fig. 6) (Waismed Limited, New York, NY, USA) – and one is re-disposable – the EZ-IO® (Fig. 7). The latter is a power driver with sealed lithium batteries enabling approximately 1000 insertions (EZ-IO® Power Driver 9050) (Teleflex, Limerick, PA, USA) or 500 insertions for the newest and smaller version (EZ-IO® G3 Power Driver 9058) without needing to charge the batteries.

When a physician practices the manual IO access procedure, he/she has to ensure a safety guard on the needle of 1 cm with thumb-index and realize an axial twisting motion to insert the needle in the bone. If the needle is inserted with the FAST®, the insertion site is located just below the sternal notch. The operator holds the introducer perpendicular to the manubrium and presses down until the needle is released. When BIG® is used, the operator holds it firmly to avoid the projection up of the needle perpendicular to the insertion site, squeezes the safety latch and pushes to eject the needle in the bone. When the needle is inserted with EZ-IO®, the operator drills the needle with the power driver into the bone perpendicular to the insertion site.

Despite a large amount of literature on the subject, there is no clear recommendation for one device over another in a given situation. In fact, comparative studies were conducted on cadaver models [18], very far from a real emergency scene, where stress is a confounding variable of performance. Furthermore, studies on patients are rarely prospective and include only a small number of patients. In one recent randomized controlled trial study comparing different devices in 107 adults, the success rate on first attempt was higher (92 % vs 83 %, *p* = 0.02) with BIG® than with EZIO® [19]. In a retrospective study of 47 IO insertions in adults between 2003 and 2010 [20], use of EZIO® was associated with higher success rate on first attempt (96 % vs 56 % or 40 %, respectively; *p* < 0.01) and higher overall success rate (96 % vs 55 % or 50 %, respectively; *p* < 0.01) compared with BIG® or manual needle. Several studies highlighted that the stylet could stick within the cannula when BIG® was used [21, 22].

### Insertion of IO devices

The procedure of IO access must be performed under sterile conditions using sterile gloves, a large sterile drape and a disposable sterile needle, after cleaning the skin to avoid causing osteomyelitis or cellulitis. There is no study comparing one antiseptic with another. In analogy with recommendations for vascular access [23, 24] or preoperative skin preparation [25], 2 % alcoholic chlorhexidine should be used in the absence of contraindications.

Different insertion sites have been evaluated, and to be safe, the following characteristics are required: a relatively thin cortical bone, a large medullary cavity, a flat surface, easy anatomical landmarks to avoid misplacement, and an easy access whatever the care provided to the patient. Three insertion sites meet these criteria in adults [26]: the proximal tibia, the distal tibia, and the proximal humerus. The sternal location has also been proposed, but the relatively thin medullary cavity leads to the risk of passing through the bone to the aorta and the heart [27]. Moreover, in the setting of anesthesia, intensive care, or emergency medicine, IO accesses are mainly used during CPR, excluding this route [26] until now. New devices for sternal use are being commercialized and will be tested [18].

The proximal tibial insertion site is located 2 cm below the tibial tuberosity, and 1 to 2 cm medial in the middle of the flat surface of the bone (Additional file 1). The distal tibial insertion site is located 2 cm superior to the medial malleolus in the middle of the flat surface of the bone (Additional file 2). The proximal humerus insertion site is located in the greater tubercle toward the coracoid process (Additional file 3). For this latter route, the arm should be flexed and internally rotated. The needle should not be inserted internally to the greater tubercle (in the *sulcus intertubercularis*) to avoid lesion to the tendon of biceps brachii.

Both the proximal tibia and the proximal humerus have high insertion success rates. The tibial location has the additional benefit of being easily affordable, even in obese patients, and of being far from the head and chest, reducing the risk of needle dislodgment during resuscitation and management of airway [28]. In a randomized controlled trial of 182 adult patients experiencing a nontraumatic out-of-hospital cardiac arrest, in which resuscitation efforts were initiated by trained paramedics, the first-attempt success rate was higher (*p* < 0.001) with tibial access (91 %, 95 % confidence interval [95 % CI]: 83–98 %) compared with either humeral access (51 %, 95 % CI: 37–65 %) or peripheral IV access (43 %, 95 % CI: 31–55 %) [6]. Median time for vascular access was shorter (*p* < 0.001) for individuals assigned to the tibial access group (4.6 min, interquartile range [IQR]: 3.6–6.2 min) compared with those assigned to the humeral access group (7.0 min, IQR: 3.9–10.0 min), and neither time was significantly different from that of the peripheral IV group (5.8 min, IQR: 4.1–8.0 min). Subsequent dislodgment was observed in 5 % (95 % CI: 0–10 %) of participants in the tibial access group compared with 20 % (95 % CI: 8–31 %) in the humeral access group and 6 % (95 % CI: 0–12 %) in the peripheral IV access group (*p* = ns). When humeral access is chosen, use of a 45-mm-long catheter increases the first-attempt success rate and immobilization of the affected arm by a strap decreases the occurrence of subsequent dislodgement [29].

Despite a lack of evidence-based medicine, administration of lidocaine in the medullary cavity has been proposed in conscious patients before injecting other drugs or fluids. Indeed, if the needle insertion is not painful, some authors describe worthier pain related to fluid administration than from traumatic injuries [30]. Yet, half of the patients receiving 20–40 mg lidocaine complain of pain during subsequent drug or fluid administration [31].

### Confirmation of correct IO device placement

Correct placement of the IO needle is confirmed by the presence of the five following signs (Table 2): sudden loss of resistance on entering the marrow cavity, ability of the needle to remain upright without support, bone marrow or blood easily sampled using a syringe, administration of 2 mL of saline without subcutaneous tissue swelling, and easy administration of fluids without resistance [6].

**Table 2:** Five signs to look for correct placement of intra-osseous devices

Loss of resistance on entering marrow cavity
Stability of the needle
Aspiration of bone marrow or blood with a syringe
Administration of 2 mL of saline without tissue swelling
Administration of 8 mL of saline without resistance

### Utility of IO access

The IO access can be used to draw blood samples. In hemodynamically stable patients, just after the IO needle is placed sodium, magnesium, calcium, lactate, glucose, blood gases (pH and PCO_2_), and hemoglobin values are similar between IO and IV accesses. Accuracy for potassium is lower but differences remained within 25 % [32, 33]. In patients with cardiac arrest, drawing blood samples is more difficult and may lead to wrong values due to low flow state and stasis in the bone. Above all, when IO access is used to infuse drugs or fluids, IO blood samples cannot be interpreted due to the dilutional effect of infused drugs and fluids. To summarize, blood samples may be obtained only in patients with spontaneous cardiac activity or during initial cardiopulmonary resuscitation before IO drug and fluid infusion [34, 35].

The IO access allows rapid fluid administration to provide the required volume resuscitation in patients in shock. The IO flow rate may reach up to 150 mL/min in either the tibial or humeral route when the pressure bag is inflated up to 300 mm Hg [36]. However, flow rates 20-fold lower have been reported with the tibial access in patients with cardiac arrest and receiving IV infusion in the homolateral femoral vein [37]. A competitive flow rate and cardiac arrest may explain these observations. Different flow rates reached with IO access according to insertion site are summarized in Table 3.

**Table 3:** Flow rate available with peripheral intravenous site according to catheter size and with intra-osseous access according to insertion site

Studies	Flow rate (mL/min)
Insyte Autogard®^*^ (Becton Dickinson, Sandy, UT, USA)	14 Gauge: 330
	16 Gauge: 193
	18 Gauge: 95
	20 Gauge: 61
Hammer et al., 2015 [15]^**^	EZ-IO tibia: 27 ± 5/69 ± 54
	EZ-IO humerus: 16 ± 3/60 ± 44
	FAST1 sternum: 53 ± 2/112 ± 47
Ong et al., 2009 [36]^**^	EZ-IO tibia: 73 ± 35/165 ± 112
	EZ-IO humerus: 84 ± 38/153 ± 65

^*^Maximum flow rate available (information provided by the manufacturer)

^**^Flow rate without/with pressure bag inflated at 300 mmHg

### Pharmacokinetics of drugs after IO administration

A variety of drugs have been delivered safely through IO access (Table 4). Theoretically, any medication that can be introduced intravenously can be introduced via an IO access. The most interesting medications during shock resuscitation are vasoactive drugs. Each drug administration should be flushed with 10 mL of fluid to rule out drug persistence in the medullar cavity [38].

**Table 4:** Key drugs and fluids usable by intra-osseous access

Adenosine
Amiodarone
Atropine
Cisatracurium
Dobutamine
Dopamine
Epinephrine
Etomidate
Heparin
Insulin
Lidocaine
Morphine
Norpinephrine
Propofol
Blood products: red blood cells/platelet/fresh frozen plasma
Resuscitative fluids: crystalloids/colloid/Ringer’s lactate
Contrast products

In a swine model of cardiac arrest, peak arterial blood concentrations of two different dye tracers were achieved faster (*p* < 0.05) for sternal IO access (53 sec) than tibial IO access (107 sec) administration during cardiopulmonary resuscitation. Moreover, delivered tibial IO dose was 65 % of sternal dose. Time to peak blood concentration and total delivered dose were similar for sternal IO and central venous administration [39]. In a recent study with a porcine septic shock model, the concentration of antibiotics was similar between IO access and peripheral IV access [40]. In the sole study in adults, pharmacokinetic parameters were similar after IO or IV administration of a single 5 mg bolus of morphine sulfate using IO access implanted in iliac crest [41].

### Securing the IO access

**Fig. 8:**
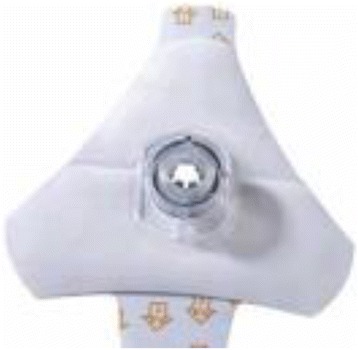
Specific adhesive tape for EZ-IO® device

To avoid misplacement due to resuscitation or inadvertent movements, the IO needle and its tubing need to be secured even if the needle stands on its own in the cortical layer. Specific adhesive tape commercialized by the devices’ manufacturers can be used (Fig. 8). Some clinicians prefer not to occlude the puncture site by an adhesive tape, because if dislodgment of the needle occurs, it will be the first place to see the subcutaneous swelling. At any rate, in both cases, the tubing must be secured with at least two omental tapes on the limb where the needle is inserted. Physicians have to make sure there is no extravasation of fluid before setting up the tape.

### Complications

Besides the inability to insert the needle or a subsequent displacement, complications of IO insertion are uncommon, lower than 1 % of insertions. Compartment syndrome due to extravasation of fluid is the most common complication. If the needle has been inserted without complications, there are no significant differences in extravasation rates between gravity and 300 mmHg pressure infusions [42]. Other serious complications include osteomyelitis, cellulitis, and skin abscesses, and all linked to prolonged IO access use [43]. Thus, the use of IO access should be limited to a few hours until IV access is achieved without exceeding 24 hours. The most serious complication leading to death was seen with sternal access. It was due to lesions to the heart or aorta and mediastinitis before specific devices for sternal puncture were available [44]. Even though microscopic pulmonary fat and marrow emboli were found in the autopsy of animals after use of IO needle insertion, fat emboli were also found in the lungs of animals with cardiopulmonary resuscitation without IO access [45]. Probably, because of the confounding factors due to the clinical situation requiring IO use, no article reports a direct involvement of IO perfusion in fat emboli syndrome in humans.

### Removing the IO device

A sterile luer lock syringe can be connected to the hub of the needle to make the hold easier. Then, with a clockwise pulling rotation, the needle can be removed and a sterile dressing applied on the puncture site, in analogy with recommendations for vascular access.

### Skill acquisition

IO access is an emergency procedure relatively easy to learn. If success rate is low for novices who have previously attended a lecture (37.5 %) without practical training, when a manual needle is used [46], it increases from 65 to 97 % when physicians use semiautomatic devices [31, 47]. After training, including a 60-min lecture introducing an algorithm for difficult vascular access with slides and videos followed by a 1-h practical session using a plastic bone model, the success rate of the EZ-IO® device on first insertion was 84 % and reached 97 % after two attempts [48]. Nevertheless, when a manual device was used, physicians could reach a high success rate (93.5 %) adopting an IO procedure assessment scale, previously validated [46], as a learning assessment tool.

In a recent study with 759 responders to a questionnaire about the nonuse of IO access when it was necessary, the main reasons for not using were lack of equipment and lack of training [13].

## Conclusions

The use of IO infusion, which was initially reserved for children, is increasing in adults, and is an alternative choice for vascular access in emergency situations today. Besides the patient in cardiac arrest, IO access is usable for patients with trauma, shock and, more globally, for every patient requiring emergency parenteral access and having difficult IV access. The contraindications are limited in number and offset by the different insertion sites available. The technique of insertion can be easily learned with high success rates after a brief training course. The IO access is therefore an indispensable tool for physicians caring for patients in life-threatening situations.

## Additional files

Additional file 1: Movie 1. Distal tibia intra-osseous insertion. (MOV 41775 kb) Additional file 2: Movie 2. Proximal tibia intra-osseous insertion. (MOV 31691 kb) Additional file 3: Movie 3. Proximal humerus intra-osseous insertion. (MOV 39491 kb)

## Abbreviations

IO: intra-osseous
IV: intravenous
